# Postburn breast reconstruction: a scoping review

**DOI:** 10.1177/20595131231202100

**Published:** 2023-09-21

**Authors:** Eduardo Gus, Jane Zhu, Stephanie G Brooks

**Affiliations:** 1 Division of Plastic, Reconstructive & Aesthetic Surgery, 7979 The Hospital for Sick Children, Toronto, Canada; 2 Department of Surgery, Temerty Faculty of Medicine, 7938 University of Toronto, Toronto, Canada; 3 Temerty Faculty of Medicine, 7938 University of Toronto, Toronto, Canada

**Keywords:** Burn, scar, reconstruction, breast, contracture, amastia

## Abstract

**Introduction:**

Postburn breast deformities pose functional and aesthetic concerns for burn patients, particularly when injury occurs before puberty, as normal breast development may be hindered. Postburn breast reconstruction aims at restoration of native anatomic features, as well as re-establishment of symmetry. The objectives of this scoping review are to map the literature on scar management and breast reconstruction, highlighting strategies that are particular to postburn deformities, as well as to establish optimal timing principles.

**Methods:**

A comprehensive search of the English literature across MEDLINE and EMBASE databases, including the grey literature, was conducted. Literature of all study designs were eligible, provided it discussed the treatment of postburn breast deformities.

**Results:**

A total of 64 studies were included. The most common study design was case series (58%) followed by retrospective cohorts (28%). Scar contracture release with split thickness skin grafts (26%) and various techniques for nipple-areola reconstruction (22%) were the most common reconstructive procedures.

**Discussion:**

Scar contracture releases predominate when there is normal breast development under a contracted skin envelope, and should be performed as soon as breast mound development is restricted. Surgical techniques widely used for postmastectomy reconstruction are required for patients with amastia or hypoplastic breasts.

**Conclusion:**

Given the heterogeneity of defects, availability of donor sites, and patient preference, no standardized guideline is available. Surgeons should combine basic scar management principles with postmastectomy techniques, adapting the surgical approach to features that are particular to thermally injured patients, as well as taking into account ideal timing considerations.

**Lay Summary:**

Breast deformities secondary to burn scars pose functional and aesthetic concerns for burn patients, particularly when injury occurs before puberty, as normal breast development may be hindered. Postburn breast reconstruction aims at restoration of native anatomic features, as well as re-establishment of symmetry. This literature review aimed at summarizing the available techniques to treat postburn breast deformities, as well as establishing optimal timing guidelines, given these issues may occur at any phase of breast development. When there is breast development under a scarred skin envelope, treatment entails scar contracture release and should be recommended as soon as the diagnosis is established, in order to allow the breast to further develop in an unrestricted manner. When there is absence of breast tissue, surgical techniques widely utilized for breast cancer reconstruction are warranted, and should be delayed until no further breast development is expected. Given the heterogeneity of deformities, availability of donor sites, and patience preference, no standardized guideline is available. Treatment options include several surgical techniques, in addition to non-surgical scar management, and timing considerations must take into account the patient's developmental phase and psychosocial wellness.

## Introduction

Thermal injury to the chest may cause significant breast deformities, particularly if the insult occurs before or during puberty, as normal breast development may be hindered. Postburn breast deformities include extrinsic scar contractures preventing the development or changing the orientation of the breast mound, obliteration of the inframammary fold (IMF), reduced breast projection, altered skin texture, and displacement, deformity or absence of the nipple-areola complex (NAC). Unilateral defects may result in significant asymmetry with the contralateral breast. Functional compromise of the breasts, such as inability to breastfeed, may occur due to lack of lactogenesis or obliteration of the lactiferous ducts. Moreover, deep injuries in the prepubertal period and burn fascial excision to the chest in any age population may engender amastia.^1–5^

The primary objective of postburn breast reconstruction is to restore the natural mammary aesthetic features, as well as symmetry of the NAC and IMF. Additional characteristics that allow for a more natural appearing breast include softness to palpation, restoration of sensitivity, mobility to accommodate in clothing, and gravity-dependent positioning.

Features that are particular to the thermally injured patient make the accomplishment of this goal challenging and require adjustments of therapeutic principles. Surgical techniques used for scar releases are typically applied for functional concerns and must be tailored to address anatomic areas of aesthetic relevance. Similarly, given the profound physiologic and anatomic deviations that burn patients may bear, such as different tissue pliability and blood supply, lack of dermis in grafted areas, and atypical fat deposits caused by the hypermetabolic response, adaptations of postmastectomy reconstruction techniques are paramount. The objectives of this scoping review are to map the literature on scar management and breast reconstruction, highlighting strategies that are particular to postburn breast deformities, as well as to establish optimal timing principles.

## Methods

We followed the Preferred Reporting of Systematic Reviews and Meta-analyses extension for Scoping Reviews (PRISMA-ScR) guideline, and Arksey and O’Malley's methodological framework.^6,7^ The protocol was registered *a priori* on Open Science Framework on October 27, 2021 (OSF Registration https://doi.org/10.17605/OSF.IO/HFP2N). With the support of a professional librarian, a comprehensive literature search was conducted across OVID MEDLINE, OVID MEDLINE Epub Ahead of Print and OVID EMBASE databases from inception to 1 November 2021. Online Supplemental Table 1 illustrates the medical subject headings included in the literature search.

All study designs were eligible for inclusion provided they reported data on individuals undergoing postburn breast reconstruction. Screening of titles and abstracts revealed potentially eligible full texts, which were retrieved and assessed. Non-English literature were excluded. Two distinct data extraction forms were generated for individual cases and aggregate data. These were modified iteratively, as permitted by scoping review methodology and with agreement from all authors.^6,7^ If a study reported both aggregate data with a few individual case reports, only the aggregate data was extracted.

The above-mentioned steps were done independently by two reviewers (SGB and JZ), and discrepancies were resolved through discussion with the help of a third reviewer (EG). The screening process was carried out on Covidence Systematic Review Software (Veritas Health Innovation, Melbourne, Australia).

## Results

**Figure 1.**
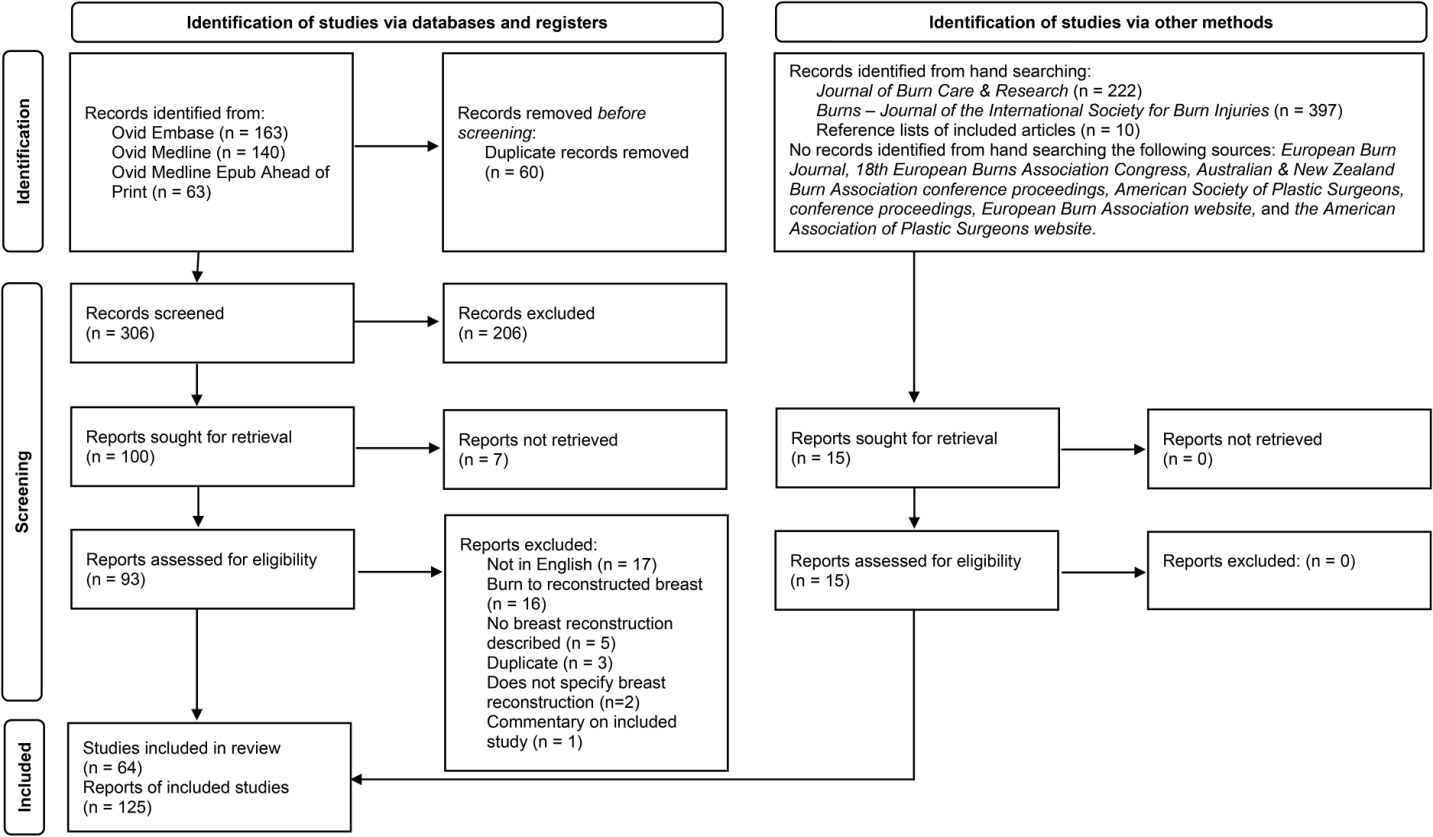
Flow diagram with included searches of databases.

After deduplication, the search strategy resulted in 306 screened studies. Of these, 206 were excluded following title and abstract screening, leaving 100 papers for full-text screening. Seven full-texts were not retrievable. Full-text screening identified a further 44 papers that did not meet inclusion criteria. Hand-searching identified a further 15 articles that were assessed for eligibility and included. A total of 64 studies were included in the scoping review (Figure 1), the majority of which were case reports/case series (58%), followed by retrospective cohorts (28%) (Table 1). Online Supplemental Table 2 contains all included studies.

**Table 1.** Overview of included studies (n = 64).

Article type	N (%)
Original article	53 (82.8)
Abstract	5 (7.8)
Editorial	3 (4.7)
Review	3 (4.7)
Study design	N (%)
Case report/case series	37 (57.8)
Retrospective cohort	18 (28.1)
Prospective cohort	4 (6.3)
Other	5 (7.8)
Year of publication	N (%)
1970–1979	6 (9.4)
1980–1989	9 (14.1)
1990–1999	5 (7.8)
2000–2009	27 (42.2)
2010–2022	17 (26.6)

**Figure 2.**
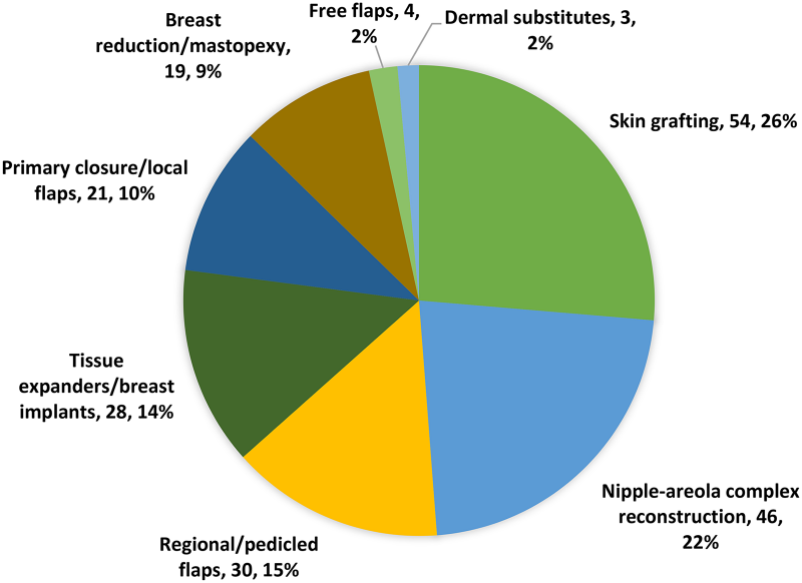
Types of reconstructive procedures.

Of the 64 included articles, incisional and excisional scar contracture release with split thickness skin grafts (STSG) (26%) and NAC reconstruction (22%) were the most common reconstructive surgical procedures reported (Figure 2). Table 2 summarizes the data extracted from individual case reports, encompassing a total of 125 patients. The majority of cases that required postburn breast reconstruction were due to flame burns (34%), followed by scalds (30%). More than half of the patients sustained a burn injury either before (47%) or during puberty (6%), and the majority underwent postburn reconstruction only after full breast development (75%). A compilation of the data extracted from case series and cohort studies can be found in Table 3.

**Table 2.** Summary of extracted data from individual case reports (n = 125).

Burn mechanism	N (%)	Timing of surgery	N (%)
Flame	43 (34.4)	Reconstruction	123 (83.7)
Scald	37 (29.6)	Acute burn care	24 (16.3)
Thermal (not specified)	12 (9.6)	Previous reconstructive state	N (%)
Friction	3 (2.4)	No previous reconstructions	60 (48.0)
Contact	1 (0.8)	1+ previous reconstructions	54 (43.2)
Electrical	1 (0.8)	Not reported	11 (8.8)
Not reported	28 (22.4)	Number of surgical interventions	N (%)
Age at burn in years	N (%)	1	58 (46.4)
0–7 (pre-pubertal)	59 (47.2)	2	45 (36.0)
8–15 (pubertal)	8 (6.4)	2+	22 (17.6)
16–39 (fully developed – young)	19 (15.2)	Complications	N (%)
40+ (fully developed – mature)	3 (2.4)	Abnormal scarring	12 (8.6)
Not reported	36 (28.8)	Aesthetic complications	8 (5.8)
Age at presentation in years	N (%)	Blood supply impairment	7 (5.0)
0–7 (pre-pubertal)	11 (7.6)	Healing impairment	5 (3.6)
8–15 (pubertal)	19 (13.2)	Issues with external devices	2 (1.4)
16–39 (fully developed – young)	98 (68.1)	None reported	103 (74.1)
40+ (fully developed – mature)	8 (5.6)		
Not reported	8 (5.6)		

**Table 3.** Summary of aggregate data.

Author, year	Study design (article type)	Sample size	Age at burn injury in years, mean ± SD (range)	Age at presentation in years, mean ± SD (range)	Surgical interventions (N)	Complications (N)
El-Otiefy et al., 2011	Retrospective cohort (original article)	55	NR	21 (13–42)	Primary closure or local flap° (NR), skin grafting (NR), NAC reconstruction (NR), breast augmentation ◊	Aesthetic complications (6), blood supply impairment (2)
Grishkevich, 2009	Case series (original article)	11	5.8 ± 3.2	19.1 ± 3.4 (15–24)	Regional flap* (NR), skin grafting (NR), breast augmentation ◊ (NR)	Blood supply impairment (2)
Guan et al.^28^	Case series (original article)	6	NR	NR (18–27)	Skin grafting (6), microsurgical flap (1), breast reduction (1), regional flap* (NR)	NR
Ivanova et al., 1977	Case series (original article)	23	NR	NR (7–23)	Primary closure of local flap° (NR), skin grafting (NR)	Blood supply impairment (7)
Levi et al., 2010	Retrospective cohort (original article)	15	NR	28, 41	Breast augmentation ◊ (30), NAC reconstruction (NR)	Issues with external devices (3), healing impairment (2), intraoperative or early postoperative (2)
McCauley et al., 1989	Retrospective cohort (original article)	28	5.9 ± 2.5	8.9 ± 2.6	Skin grafting (18), regional flap* (2)	NR
Motamed et al., 2005	Case series (original article)	6	NR [child]	20 ± 3	NAC reconstruction (6), skin grafting (NR)	NR
Mudge et al., 2000	Retrospective cohort (abstract)	6	2.4 ± 1.3	19.2 ± 1.6	Breast reduction (6)	NR
Neale et al., 1982	Retrospective cohort (original article)	157	NR	14 (11–18)	NAC reconstruction (64), primary closure or local flap° (NR), regional flap* (NR), breast augmentation ◊ (NR)	NR
Ozgur et al., 1992	Case series (original article)	24	NR [child]	NR (13–34)	Primary closure or local flap° (4), regional flap* (6), skin grafting (6), NAC reconstruction (3), breast augmentation ◊ (8)	NR
Pakhomov et al., 1984	Case series (abstract)	27	NR	NR (12–27)	Microsurgical flap (9), breast augmentation ◊ (7), primary closure or local flap (5)	NR
Palao et al., 2003	Retrospective cohort (original article)	12	NR [child]	NR (12–27)	Skin grafting (11), dermal substitute♦ (11), breast augmentation ◊ (1), primary closure or local flap° (1), regional flap* (1)	Aesthetic complications (12), blood supply impairment (2), abnormal scarring (2), intraoperative or early postoperative (1)
Pensler et al., 1986	Retrospective cohort (original article)	84	NR	16 ± 1.2 (13–18)	NAC reconstruction (84)	Aesthetic complications (15), abnormal scarring (1)
Psillakis et al., 1985	Case series (original article)	2	NR	NR	Regional flap* (2), breast reduction (NR)	NR
Sadeq et al., 2020	Retrospective cohort (original article)	96	6.4 ± 4.8	NR	Skin grafting (48), primary closure or local flap° (43), NAC reconstruction (15), breast reduction (8), breast augmentation ◊ (4), regional flap* (2)	NR
Sadove et al., 2005	Retrospective cohort (original article)	2	NR	15	Skin grafting (NR), NAC reconstruction (NR), breast reduction (NR)	NR
Sakr, 2002	Retrospective cohort (original article)	36	12.16	24.6 (16–48)	Breast augmentation ◊ (1), primary closure or local flap° (NR), skin grafting (NR)	Abnormal scarring (NR)
Tredget et al., 2019	Retrospective cohort (abstract)	NR	NR	NR	Free flap (NR)	NR
van Straalen et al., 2000	Retrospective cohort (original article)	15	NR	NR	NAC reconstruction (15)	Intraoperative or early postoperative (1)
Zhernov, 2009	Retrospective cohort (original article)	9	NR	NR	Breast augmentation ◊ (20), primary closure or local flap° (6)	NR

NAC = nipple areola complex.

° Z-plasty, W-plasty, transposition flap.

* Latissimus dorsi muscle flap, pectoralis muscle flap, reverse abdominoplasty, local fasciocutaneous flap, transverse rectus abdominis muscle flap, thoracodorsal artery perforator fasciocutaneous flap.

♦Biodegradable Temporising Matrix, Integra.

◊ Tissue expanders, breast implants, autologous augmentation.

## Discussion

Postburn breast reconstruction can be challenging even to the experienced surgeon. Patients can have a myriad of deformities, with varying degrees of severity, linked to either the skin envelope and/or the breast parenchyma. In the context of bilaterality, this entails symmetry issues. As thermal injury can occur at any age, while breasts do not develop until puberty, and change over time with aging and breastfeeding, there is also a significant timing component associated with surgical decision-making.

We anticipated that given the multitude of defects and treatment options, no rigorous guidelines would be available to dictate surgical approach, and we conducted a scoping review to map the literature on this topic. As opposed to systematic reviews, which respond to narrow research questions and focus on the quality of the studies, this knowledge synthesis approach centers on capturing a wide range of study designs, in order to gather all available literature on the topic at hand.^6–9^ Although the main focus of the scoping review was long-term breast reconstruction, we included a few studies containing acute burn care features that could be impactful for the long-term outcomes of this population. The current body of literature on postburn breast reconstruction comprises mainly of case reports, case series and cohort studies in which authors describe using the principles of scar releasing and postmastectomy breast reconstruction, highlighting concerns and strategies they used to address features that are particular to the thermally injured.

While we found that thermal injury occurred before or during puberty in approximately 53% of the cases reported in the literature, Sadeq et al. noted that 83% of their 96-patient cohort sustained burn injury in the prepubescent period.^10^ Pediatric scald injuries to the chest typically affect the skin only; however, deep scalds and flame injuries may involve the breast bud, which is located beneath the NAC.^4^ In such instances, conservative acute treatment of the burned NAC is recommended because of its remarkable ability to regenerate from the epithelium of the lactiferous ducts.^4,11,12^ Even full thickness injuries to the NAC may be treated conservatively to allow for spontaneous eschar separation and healing by secondary intent, which results in better outcomes than surgical reconstruction. Furthermore, as the breast bud is hidden deep into the subcutaneous tissue, breast mound development may ensue later in puberty even in the absence of the NAC.^1,4,5,10,13–17^ As sheet grafts contract less than meshed grafts and result in better skin texture, they are preferred in the acute care setting.^18,19^

When scars are limited to the skin and the breast bud is spared, the pubertal breast parenchyma will develop underneath a contracted skin envelope, leading to deformity. The dogma of surgically intervening on breasts only once full breast development is achieved may explain why most patients sustain burn injuries before or during puberty, only to undergo reconstruction later in life. Most authors agree that breast releases should be performed as soon as breast tissue shows signs of bulging over non-scarred areas or when early breast mound development is clearly restricted by inelastic scar tissue.^1,2,11,17,19–22^ This strategy aims at correcting the original extrinsic defect, allowing the breast to develop in a natural manner. Moreover, as the tissue within the scarred, inelastic torso grows, ventilation may become compromised in patients with severely contracted breasts.^23^ In addition to physical considerations, timing of surgical intervention should be tailored according to the psychosocial and sexual development of the patient. As most injuries occur during childhood, long-term periodic follow-up of pediatric patients with thermal injury to the chest is warranted to optimize timely intervention.^4^

All currently available scar management techniques may provide benefits to postburn breast deformities, particularly when the damage is restricted to the skin envelope. These include compression garments, silicone gel, laser treatment, Z-plasties, transposition flaps and their variations, as well as incisional and excisional (resurfacing) contracture releases. Details on these procedures can be found in the general burn reconstruction literature and are beyond the scope of this review. The general principle of recommending non-surgical scar management techniques before surgical scar releases—to reduce fibroblast activity and increase tissue pliability—also applies to this population.^16,24,25^

Common incisional scar contracture releases to the breasts include reconstruction of the IMF by means of a U-shaped release of the inferior pole with placement of a thick split thickness or full thickness skin graft. Another common incisional release associated with postburn breast deformity is to the superolateral aspect of the breast, to separate the mound from shoulder and axilla. In patients with bilateral breast contractures, an inverted T- or Y-shaped incisional contracture release over the sternum may be required.^25,26^ Excisional release with reshaping of the breast parenchyma to achieve a round contour and higher projection, as well as resurfacing with thick STSG has been reported.^21,27^ Local skin and fasciocutaneous flaps from the upper chest, shoulders, flanks and upper abdomen have been suggested to provide better color match, and re-contract less often than skin grafts.^29^ Tissue expanders in adjacent anatomic areas have been used to augment the available flap size.^30–32^ For defects to the lower pole, Kalender et al. described the use of a contralateral fasciocutaneous island flap based on the internal mammary perforators, hiding the donor site scar in the IMF.^33^ A modified transverse rectus abdominis myocutaneous (TRAM) flap utilizing an island of upper abdominal skin based on the superior epigastric vessels that includes the IMF and lower pole of the contralateral breast has been reportedly used to reconstruct contracted lower poles and elevate the NAC.^34^ Free flaps have been proposed to release scars due to their lower chance of re-contracture. Cutaneous and fasciocutaneous designs, such as the anterolateral thigh (ALT), radial forearm, deep inferior epigastric perforator (DIEP), and ulnar artery flaps have been reported.^35,36^ Most authors agree that in cases of concurrent extrinsic scar contracture and intrinsic breast deformities or hypoplasia, extrinsic releases should precede any intrinsic breast procedure to decrease the tension onto the breast mound scars.^1,3,4,37^

The use of dermal matrices has been attempted to primarily prevent scar contractures and to prevent their recurrence once breast scars are released. Palao et al. reported the use of Integra Dermal Regeneration Template (Integra LifeSciences Corporation, US) to perform resurfacing of the breasts with very thin STSG (0.005 inch).^38^ Tsoutsos and colleagues performed breast reconstruction combining the use of tissue expanders and Integra to resurface the breast, reporting good expansion of the resurfaced area.^39^ The use of mesenchymal stem cell-enriched Integra to resurface the breasts has also been proposed.^40^ More recently, Telianidis et al. constructed a bra using NovoSorb Biodegradable Temporising Matrix (Polynovo, Australia) in the acute burn setting, grafting bilateral breasts five weeks after template application, which allowed them to use meshed grafts.^41^

As major burn survival rates have significantly increased, many patients who require postburn chest reconstruction present with severely scarred or contracted breast footprints, or even amastia. The complete absence of breast tissue may ensue after a fourth-degree burn, by direct damage to the breast bud in the prepubertal period, or by fascial excision of deep burns to the anterior chest in any age population. Treatment of pubertal individuals with suspected postburn amastia must include a conservative period of observation to ensure breast development is absent as opposed to delayed.^1^

For cases with confirmed amastia or hypoplasia, bulky pedicled and free flaps are potential reconstructive options. Extensive expertise with flap designs extrapolated from postmastectomy procedures has engendered excellent outcomes for postburn deformities; however, adaptations are often required, as conventional flap designs may not be available due to scarred donor sites. Moreover, body habitus alterations due to the profound metabolic derangements in major burn survivors may lead to variations in angiosome areas and subcutaneous volume. For perforator and free flaps, sonography with a handheld doppler and/or CT angiogram have been recommended to mitigate risks associated with poor blood supply.^42^ Popular abdominal flaps include the TRAM and DIEP flaps; however, lack of skin laxity and scars to the lower abdomen may prevent their use.^4,22,43^ Other options reported include a vertically-oriented thoracodorsal artery perforator (TDAP) flap,^2^ transverse myocutaneous gracilis flap,^44,45^ and unconventional flap designs given local deformities, such as the one reported by Boehm et al., which combines a tensor fascia lata with ALT flap.^42^

For patients with no available autologous tissue transfer options or who prefer to be treated with alloplastic techniques, tissue expanders are usually necessary for the creation of pockets where the breast implants will later be placed. Compared to pristine skin, scarred skin has increased resistance and greater instability; hence, expansion must be done at a slower pace, and submuscular pockets are preferred. Nonetheless, tissue expanders have been suggested to act as internal compression devices, possibly adding benefit as scars softeners.^10,17,19,31,43,46–48^ In patients with thick scar tissue that prevents the expander to fully inflate, radial stellate-shaped incisional contracture release to the breast mound has been reported to be effective.^49^ Endoscopic alloplastic techniques have reportedly shown lower incidence of complications, which is likely associated with the remote placement of incisions.^24,47^ The combination of implant-based reconstruction with latissimus dorsi myocutaneous flaps has been suggested to avoid the expansion phase, as well as to create a permanent and sharp IMF.^15,20^

Modifications of aesthetic breast procedures and their combination with scar contracture releases to treat post burn deformities date as early as 1977.^50–52^ Hsiao et al. reported combined mammary augmentation with incisional scar releases in one-stage procedures.^46^ Hunter et al. reported the excision of superomedial breast parenchyma and hypertrophic scars, combined with a rotation mammoplasty to bring pristine skin from the lower to the upper pole.^53^ Gheita et al. utilized de-epithelialized tissue from the distal aspect of a reverse abdominoplasty flap to augment a breast with postburn defect.^47^ The lateral rotation of the axis of a conventional vertical mammoplasty design has been reported to medialize the NAC and excise lateral scarred skin.^31,54^ There are numerous cases of improving breast symmetry by means of a balancing mastopexy or breast reduction,^1,4,10,19,53,55,56^ as well as liposuction and fat grafting.^42,44,47^ Furthermore, there are reports of utilizing the full-thickness skin paddle that would be otherwise discarded from breast reductions and abdominoplasties for scar releases.^43,57^

NAC reconstruction should only take place after breast scars are settled and mature, usually 9–12 months after breast mound reconstruction. Breasts that are released prior to full breast development should only undergo NAC reconstruction after full growth has occurred.^17,19,20^ Full thickness skin grafts from inner thighs, labia minora and scrotum usually develop hyperpigmentation and have been used for areolar reconstruction. Medical tattooing is often recommended to mitigate residual hypopigmentation and irregular circumferential scars. Composite grafts from the earlobe, nipple-sharing techniques, and local skin flaps are established options for nipple reconstruction.^1,3,10,20,22,58–61^ However, given the constant radial pull that scar tissue exerts onto the central breast mound, conventional NAC reconstruction techniques can have limited success. These forces often enlarge areolas reconstructed with skin grafts, and flatten the nipples constructed by local flaps. In addition, subdermal blood supply may be interrupted by scar tissue, which increases ischemic complications to local flaps. To overcome these unique problems, Bunchman et al. have proposed the “Double Bubble” technique, in which two concentric incisions are made, one around the neo-areola, and the other around the neo-nipple. The skin edges are then sutured to the subcutaneous tissue, which is left open to heal by secondary intention, purposefully creating circumferential scar contractures that elevate the central areas, resembling natural areola and nipple.^62^ Caviggioli et al. have used fat grafting to restore projection of nipples flattened by mature burn scars, obtaining a mean projection of 2.9 mm at 2-year follow-up.^63^ For patients with existing but displaced NACs, Mohmand et al. described a modification of the classic Z-plasty technique, in which the flaps are U-shaped as opposed to triangular, and one of them contains the NAC.^64^ van Straalen published on the transposition of two subcutaneous-pedicled flaps, one of which contains the NAC, reporting good patient satisfaction.^65^

We found only one report of acute burn reconstruction to lactating breasts. Despite being a very specific patient population, it is important to disseminate that no surgical treatment – either acute or long-term reconstruction – should take place prior to termination of lactation and glandular involution. Particular risks associated with lactating breasts include mastitis, engorgement, milk fistulae, and glandular loss.^66^

Despite endeavoring to perform a thorough literature search, this scoping review is limited to literature published in the English language. In addition, in the absence of experimental research designs, most of the information provided was collected from case reports, case series and cohort studies, and may reflect the source author's opinions and practices, rather than objective evidence. Moreover, we found no epidemiological data on breast burn sequelae. This may be related to the fact that there is a significant time lapse between acute burn management, frequently in childhood, and the pursuit of breast reconstruction, which mostly occurs years later, engendering loss of follow up. Nevertheless, to our knowledge this is the first scoping review to map the literature on postburn breast reconstruction, highlighting the caveats that are unique to the thermally injured. In addition to providing technical details, this review mitigates the incorrect dogma of postponing all interventions to once full breast development has occurred. Timing of intervention must be individually tailored considering physical, developmental, and psychological aspects (Table 4).

**Table 4.** Summary of timing considerations.

Deformity feature	Timing of intervention
Presence of breast mound development, with entrapment underneath a contracted skin envelope	EARLY intervention, in an attempt to allow the breast mound to develop naturally.
Amastia; lack of breast development	CONSERVATIVE approach to confirm breast development is absent, as opposed to delayed. Intervene only AFTER contralateral breast is fully developed, to maximize symmetry.
Association of extrinsic and intrinsic scar contractures; or extrinsic scar contractures and amastia	Treat extrinsic scar contractures BEFORE intrinsic ones or amastia.
Absence or displacement of NAC	Perform NAC reconstruction/repositioning 9–12 months after mound reconstruction, to allow for preceding scar maturation. For breast mounds that were released during development, postpone NAC reconstruction to after full breast development.
*Psychosocial and sexual development features must be taken into account, and may precipitate or postpone surgical interventions.*	

## Conclusion

Thermal injury may cause significant breast deformities with associated functional, aesthetic, and psychological morbidity. Given the significant variability of defects resulting from burn scars, the availability of donor sites, and patient preference, no standardized guideline is universally applicable. Surgeons should follow basic burn reconstruction principles and rely on breast reconstruction techniques widely used for postmastectomy defects, taking into account the features that are unique to burn survivors. Moreover, timing considerations are paramount for a successful postburn breast reconstruction, particularly for patients who sustained thermal injury prepubertally. Long-term periodic follow-up may be required to ensure pediatric patients sustaining chest burns undergo proper breast development and timely breast reconstruction.

## Supplemental Material

sj-docx-1-sbh-10.1177_20595131231202100 - Supplemental material for Postburn breast reconstruction: a scoping review Click here for additional data file. Supplemental material, sj-docx-1-sbh-10.1177_20595131231202100 for Postburn breast reconstruction: a scoping review by Eduardo Gus, Jane Zhu and Stephanie G Brooks in Scars, Burns & Healing

sj-docx-2-sbh-10.1177_20595131231202100 - Supplemental material for Postburn breast reconstruction: a scoping review Click here for additional data file. Supplemental material, sj-docx-2-sbh-10.1177_20595131231202100 for Postburn breast reconstruction: a scoping review by Eduardo Gus, Jane Zhu and Stephanie G Brooks in Scars, Burns & Healing
