# Supplementary material for: Postburn breast reconstruction: a scoping review
Source: Scars Burn Heal. 2023 Sep 21;9:20595131231202100. doi: 10.1177/20595131231202100 (PMC10512695; doi:10.1177/20595131231202100)
Supplement: sj-docx-1-sbh-10.1177_20595131231202100 - Supplemental material for Postburn breast reconstruction: a scoping review [file sj-docx-1-sbh-10.1177_20595131231202100.docx]

Supplemental Table 1. Ovid MEDLINE Search Strategy

| **Row** | **Searches** | **Results** |
| --- | --- | --- |
| 1 | burns/ or burns, chemical/ or burns, electric/ or sunburn/ | 57884 |
| 2 | (scald or scalds).ti,ab,kf. | 2463 |
| 3 | 1 or 2 | 58311 |
| 4 | breast/ or nipples/ | 47068 |
| 5 | areola*.ti,ab,kf. | 3815 |
| 6 | 4 or 5 | 48765 |
| 7 | 3 and 6 | 140 |
