# Supplementary material for: Postburn breast reconstruction: a scoping review
Source: Scars Burn Heal. 2023 Sep 21;9:20595131231202100. doi: 10.1177/20595131231202100 (PMC10512695; doi:10.1177/20595131231202100)
Supplement: sj-docx-2-sbh-10.1177_20595131231202100 - Supplemental material for Postburn breast reconstruction: a scoping review [file sj-docx-2-sbh-10.1177_20595131231202100.docx]

Supplemental Table 2. Included articles

|  | **Author** | **Year** | **Article title** | **Article type** | **Study design** |
| --- | --- | --- | --- | --- | --- |
| 1 | Acartürk et al | 2020 | Total aesthetic reconstruction of postburn bilateral breast loss using transverse myocutaneous gracilis free flaps: a case report and literature review | Original article | Case report |
| 2 | Al-Qattan el at | 1994 | Management of acute burns of the female pediatric breast: delayed tangential excision versus spontaneous eschar separation | Original article | Case report |
| 3 | Arkoulis et al | 2018 | The Role of adipose derived stem cell enhanced dermal scaffolds in the treatment of chronic burns contractures | Abstract | Prospective cohort |
| 4 | Bayram et al | 2014 | Custom-made approach to a patient with post-burn breast deformity | Original article | Case report |
| 5 | Bishop et al | 1979 | The burned female breast | Original article | Case report |
| 6 | Boehm et al | 2018 | Autologous breast reconstruction using a tensor fascia lata/anterior lateral thigh-freestyle flap after extensive electric burn: a case report | Original article | Case report |
| 7 | Bunchman et al | 1974 | Nipple and areola reconstruction in the burned breast - the 'double bubble' technique | Original article | Case report |
| 8 | Cartotto et al | 2014 | Common postburn deformities and their management | Review | N/A |
| 9 | Caviggioli et al | 2010 | Nipple resuscitation by lipostructure in burn sequelae and scar retraction | Editorial | Prospective cohort |
| 10 | DiPirro | 1970 | Reconstruction of the nipple and areola after a burn - case report | Original article | Case report |
| 11 | Ebrahiem et al | 2019 | Inferior pole breast reconstruction by TDAP flap in post-burn breast contracture | Original article | Retrospective cohort |
| 12 | Ebrahiem et al | 2019 | Inferior pole breast reconstruction by TDAP flap in post-burn breast contracture | Original article | Retrospective cohort |
| 13 | El-Ptiefy et al | 2011 | Post-burn breast deformity: various corrective techniques | Original article | Retrospective cohort |
| 14 | Erol et al | 1982 | Areola transposition technique in the reconstruction of breast deformities due to burns | Original article | Case report |
| 15 | Foley et al | 2008 | Breast burns are not benign: long-term outcomes of burns to the breast in pre-pubertal girls | Original article | Retrospective cohort |
| 16 | Gheita et al | 2014 | Strategies in the management of post-burn breast deformities | Original article | Retrospective cohort |
| 17 | Gokrem et al | 2003 | Correction of a mild breast contracture with a new technique:“V-Y-Z-PLASTY” | Original article | Case report |
| 18 | Graham et al | 1976 | Chest burns in the young girl | Editorial | N/A |
| 19 | Grishkevich | 2009 | Restoration of the shape, location and skin of the severe burn-damaged breast. | Original article | Case series |
| 20 | Guan et al | 1988 | Reconstruction of postburn female breast deformity | Original article | Case series |
| 21 | Haik et al | 2007 | Expanded reverse abdominoplasty for reconstruction of burns in the epigastric region and the inframammary fold in female patients | Original article | Case report |
| 22 | Giele et al | 1994 | Management of full thickness burns to lactating breasts | Original article | Case report |
| 23 | Hsiao et al | 2009 | Are augmentation mammaplasty and reconstruction of the burned breast collateral lines? Experience in performing simultaneous reconstructive and aesthetic surgery | Original article | Prospective cohort |
| 24 | Hunter et al | 2009 | Correction of postburn superior pole breast deformity and macromastia--a novel approach | Original article | Case report |
| 25 | Ivanova et al | 1977 | Surgical repairment of mammary gland scar deformities caused by burn injuries | Original article | Case series |
| 26 | Jargis et al | 2016 | Release and reconstruction of a postburn deformed breast in a young woman | Original article | Case report |
| 27 | Kalendar et al | 1999 | Breast reconstruction with the internal mammary artery pedicled fasciocutaneous island flap: description of a new flap | Original article | Case report |
| 28 | Kunert et al | 1988 | Principles and procedures in female breast reconstruction in the young child's burn injury | Original article | Case report |
| 29 | Levi et al | 2010 | A comparative analysis of tissue expander reconstruction of burned and unburned chest and breasts using endoscopic and open techniques | Original article | Case series |
| 30 | Livaoğlu et al | 2008 | Reconstruction of a burned breast with vertical mammaplasty: a case report | Original article | Case report |
| 31 | Loss et al | 2002 | The burned female breast: a report on four cases | Original article | Case report |
| 32 | MacLennan et al | 2000 | Reconstruction of the burned breast | Original article | Retrospective cohort |
| 33 | McCauley et al | 1989 | Longitudinal assessment of breast development in adolescent female patients with burns involving the nipple-areolar complex | Original article | Retrospective cohort |
| 34 | Mohamed et al | 2017 | Treatment of extensive post-burn deformities using extra-large sheets of full thickness skin grafts | Original article | Case report |
| 35 | Mohmand et al | 2002 | Double U-plasty for correction of geometric malposition of the nipple-areola complex | Original article | Case report |
| 36 | Monteiro et al | 2002 | Tissue expansion in reconstruction of the burned breast | Original article | Case report |
| 37 | Motamed et al | 2005 | Postburn reconstruction of nipple-areola complex | Original article | Case series |
| 38 | Mudge et al | 2000 | Correction of asymmetry in the unilaterally burned breast | Abstract | Retrospective cohort |
| 39 | Mueller et al | 2002 | Post-burn breast resurfacing using an abdominal full-thickness skin graft | Original article | Case report |
| 40 | Neale et al | 1982 | Breast reconstruction in the burned adolescent female (an 11-year, 157 patient experience) | Original article | Retrospective cohort |
| 41 | Ogilvie et al | 2008 | Burns of the developing breast | Review | N/A |
| 42 | Ozgenel et al | 2002 | Reconstruction of burn-damaged female breasts | Original article | Retrospective cohort |
| 43 | Ozgur et al | 1992 | Reconstruction of postburn breast deformities | Original article | Case series |
| 44 | Pakhomov et al | 1984 | Scar deformities of the mammary glands – Surgical treatment | Abstract | Case series |
| 45 | Palao et al | 2003 | Burned breast reconstructive surgery with Integra dermal regeneration template | Original article | Retrospective cohort |
| 46 | Payne et al | 2003 | Correction of postburn breast asymmetry using the LeJour-type mammaplasty technique | Original article | Case report |
| 47 | Pensler et al | 1986 | Reconstruction of the burned nipple-areola complex | Original article | Retrospective cohort |
| 48 | Psillakis et al | 1985 | Burned breast: Treatment with a transverse rectus abdominis island musculocutaneous flap | Original article | Case series |
| 49 | Sadeq et al | 2020 | Reconstruction of severe burns to the breast in pediatric patients: A 10-year experience | Original article | Retrospective cohort |
| 50 | Sadove et al | 2005 | Congenital and acquired pediatric breast anomalies: a review of 20 years' experience | Original article | Retrospective cohort |
| 51 | Sakr | 2002 | Local fascicutaneus flaps in managing post burn breast deformities | Original article | Retrospective cohort |
| 52 | Sawhney et al | 1977 | The correction of post-burn contractures of the breast | Editorial | N/A |
| 53 | Sharriff et al | 2007 | Burned breast reconstruction by expanded artificial dermal substitute | Original article | Case report |
| 54 | Shelley et al | 2006 | Dual benefit procedures: combining aesthetic surgery with burn reconstruction | Original article | Case report |
| 55 | Slator et al | 1991 | Postburn breast reconstruction: tissue expansion prior to contracture release | Original article | Case report |
| 56 | Telianidis et al | 2020 | Case report: the use of biodegradable temporising matrix in breast reconstruction following flame burn to chest | Original article | Case report |
| 57 | Tredget et al | 2019 | The Advantages of Fasciocutaneous Free Tissue Transfers for the Management of Post-Burn Scar Contractures | Abstract | Retrospective cohort |
| 58 | Tsai et al | 2004 | Free split-cutaneous perforator flaps procured using a three-dimensional harvest technique for the reconstruction of postburn contracture defects | Original article | Case report |
| 59 | Tsoutsos et al | 2007 | Burned breast reconstruction by expanded artificial dermal substitute | Original article | Case report |
| 60 | van Straalen et al | 2005 | Correction of the post-burn malpositioned nipple-areola complex by transposition of two subcutaneous pedicled flaps | Original article | Retrospective cohort |
| 61 | van Aalst et al | 2009 | Pediatric chest wall and breast deformities | Review | N/A |
| 62 | Versaci et al | 1986 | Breast reconstruction by tissue expansion for congenital and burn deformities | Original article | Prospective cohort |
| 63 | Weitgasser et al | 2018 | A rare approach? Microsurgical breast reconstruction after severe burn | Original article | Case report |
| 64 | Zhernov | 2009 | Use of modified flaps in chest and breast reconstruction | Abstract | Retrospective cohort |
